# Incidence and influencing factors of kinesiophobia in patients with chronic heart failure: a scoping review

**DOI:** 10.3389/fpsyg.2024.1395199

**Published:** 2024-07-31

**Authors:** Qin Xiang, Xiao-Yun Xiong, Mei-Jun Zhang, Si Liu, Hua Chen, Meng-Die Liu, Ying Wang, Ying Yang

**Affiliations:** ^1^Department of Nursing, The 2nd Affiliated Hospital, Jiangxi Medical College, Nanchang University, Nanchang, China; ^2^School of Nursing, Jiangxi Medical College, Nanchang University, Nanchang, China

**Keywords:** Kinesiophobia, chronic heart failure, cardiac rehabilitation, influencing factors, mental health, scoping review

## Abstract

**Introduction:**

Kinesiophobia denotes an excessive and irrational apprehension towards physical activity or exercise among patients, stemming from a perception of susceptibility to painful injury or re-injury. Cardiac rehabilitation stands pivotal in the secondary prevention spectrum for individuals with cardiovascular ailments, with exercise constituting a cornerstone of this regimen. However, the emergence of kinesiophobia poses a formidable challenge, diminishing patient adherence to cardiac rehabilitation protocols, particularly among those grappling with chronic heart failure. To bolster exercise-based rehabilitation initiatives in this cohort, a thorough comprehension of the multifaceted factors precipitating kinesiophobia is imperative. This review endeavors to delineate prevailing evidence and prevalence concerning kinesiophobia triggers in chronic heart failure patients, while pinpointing research lacunae for future exploration.

**Methods:**

Employing a scoping review methodology, our investigation culled data from diverse scholarly databases, including Embase, PubMed, Scopus, CINAHL, Web of Science, Medline, Sinomed, CNKI, Wangfan, and VIP.

**Results:**

After thorough evaluation, 9 studies that met the inclusion criteria were ultimately incorporated.

**Discussion:**

Our findings underscore a notable prevalence of kinesiophobia in chronic heart failure patients, predominantly influenced by socio-demographic factors, psychological and cognitive factors, disease and treatment factors, as well as lifestyle and behavior. Armed with these insights, future interventions can be tailored to mitigate kinesiophobia levels, fostering enhanced engagement in exercise-centric cardiac rehabilitation endeavors among patients grappling with chronic heart failure.

## 1 Introduction

Cardiovascular disease is a major public health problem worldwide, characterized by high morbidity and high mortality, and is the leading killer of human health (Benjamin et al., 2019), which will increase the heavy burden on individuals and social medical and health service system (Cook et al., 2014; The Writing Committee Of China, 2023). More than 64 million people worldwide suffer from heart failure (Savarese et al., 2023). Cardiac exercise rehabilitation originated in the 1960s (Clausen et al., 1969), which is a comprehensive rehabilitation strategy consisting of five prescriptions including drugs, exercise, nutrition, psychology (including sleep management), smoking cessation and alcohol restriction. Many guidelines recommend exercise rehabilitation for patients with cardiovascular disease (Ponikowski et al., 2016; Sharma et al., 2021). For example, the ‘Ten Commandments’ for the 2020 ESC Guidelines on sports cardiology and exercise in patients with cardiovascular disease clearly state that “All able individuals with cardiovascular disease should engage in at least 150 min of exercise per week divided over 5 days, or 75 min of vigorous exercise divided over 3 days” (Sharma et al., 2021). Kinesiophobia is defined as an excessive, irrational sense of vulnerability to sports or daily activities due to a reduced threshold of the body to pain due to injury such as pain (Vlaeyen et al., 1995), it is also known as fear of movement, and so on. Changes in cardiac structure and decreased cardiac output in heart failure patients contribute to respiratory muscle atrophy, exacerbating dyspnea (Nakagawa et al., 2020). Concurrently, pulmonary congestion and infection further compromise lung function, resulting in symptoms such as coughing and expectoration (Douillet et al., 2023). These combined factors significantly diminish the quality of life for affected patients. Kinesiophobia can exacerbate dyspnea and lead to discomfort such as chest tightness and palpitations. Furthermore, it can increase psychological stress and negative emotions in patients (Qin J-. W. et al., 2022; Tang et al., 2023), reducing treatment compliance and posing a major obstacle to participation in exercise-based cardiac rehabilitation for heart failure patients (Rogerson et al., 2012). Exercise rehabilitation has been proven to be an effective secondary prevention measure for patients with Chronic Heart Failure (CHF). It serves as a cornerstone and crucial component of cardiac rehabilitation, offering numerous benefits to patients, as evidenced by studies (Pelliccia et al., 2021; Youn et al., 2023). Studies have shown that kinesiophobia is one of the key factors for non-adherence to home-based cardiac rehabilitation in patients with chronic heart failure (Yang et al., 2023a,b). Despite the growing body of research on kinesiophobia, there is a lack of systematic reviews that have comprehensively outlined the influencing factors of kinesiophobia in patients with chronic heart failure. Therefore, the aim of this commentary is to delineate the current influencing factors of kinesiophobia in patients with chronic heart failure and identify potential research gaps to address future research needs in this area. Our review addresses two key research questions: (a) What is the prevalence of kinesiophobia in patients with chronic heart failure? (b) What are the factors that influence kinesiophobia in patients with chronic heart failure?

## 2 Methods

While we applied the scoping review methodology proposed by Arksey and O'Malley (2005) and advanced by Levac et al. (2010), we did not perform the sixth step, i.e., consultation, because we examined the study related to the influencing factors of kinesiophobia in patients with chronic heart failure, we did not address the perspectives of other stakeholders on this issue.

### 2.1 Identifying the research question

The purpose of this review is to find the existing study on the influencing factors of kinesiophobia in patients with chronic heart failure and the research gaps in the study. To achieve these goals, we asked the following research questions: (a) What is the incidence of kinesiophobia in patients with chronic heart failure? (b) What are the influencing factors of kinesiophobia in patients with chronic heart failure?

### 2.2 Identifying relevant studies

Two investigators with extensive evidence-based knowledge participated in the systematic review search, which involved multiple searches of the following databases: PubMed, Scopus, CINAHL, Web of Science, Embase, Medline, Sinomed, CNKI, Wangfan, VIP. The selection of search terms was initially established through consultations with experts and discussions among members of the subject group. Following a trial search, the following search terms were finalized: heart failure/CHF/chronic heart failure/cardiac failure*/heart decompensation/right-side heart failure/myocardial failure/congestive heart failure/left-side heart failure/kinesiophobia/fear of movement/fear of physical activity/risk factors/relative risk/social risk factor*/health correlate*/population at risk/risk score/risk factor score*/cause/protective factor. The search period was from the establishment of the database to December 20, 2023. Languages are limited to English and Chinese. After the search was completed, we read the reference lists of each article and found no new relevant articles. In addition, we discussed and identified 4 common core journals. Keywords in these journals were manually searched to identify missing study, and no new study was found. The search strategy is described in the Appendix 1.

### 2.3 Study selection

Our study inclusion criteria encompassed studies involving individuals diagnosed with chronic heart failure, focusing on elucidating the factors influencing the development of kinesiophobia within this population. Accepted study designs include qualitative, quantitative, mixed, or multimethod approaches. The exclusion criteria comprised conference abstracts, editorials, letters, studies lacking access to full-text study, and duplicate publications.

### 2.4 Study screening and data extraction and analysis

**Figure 1:**
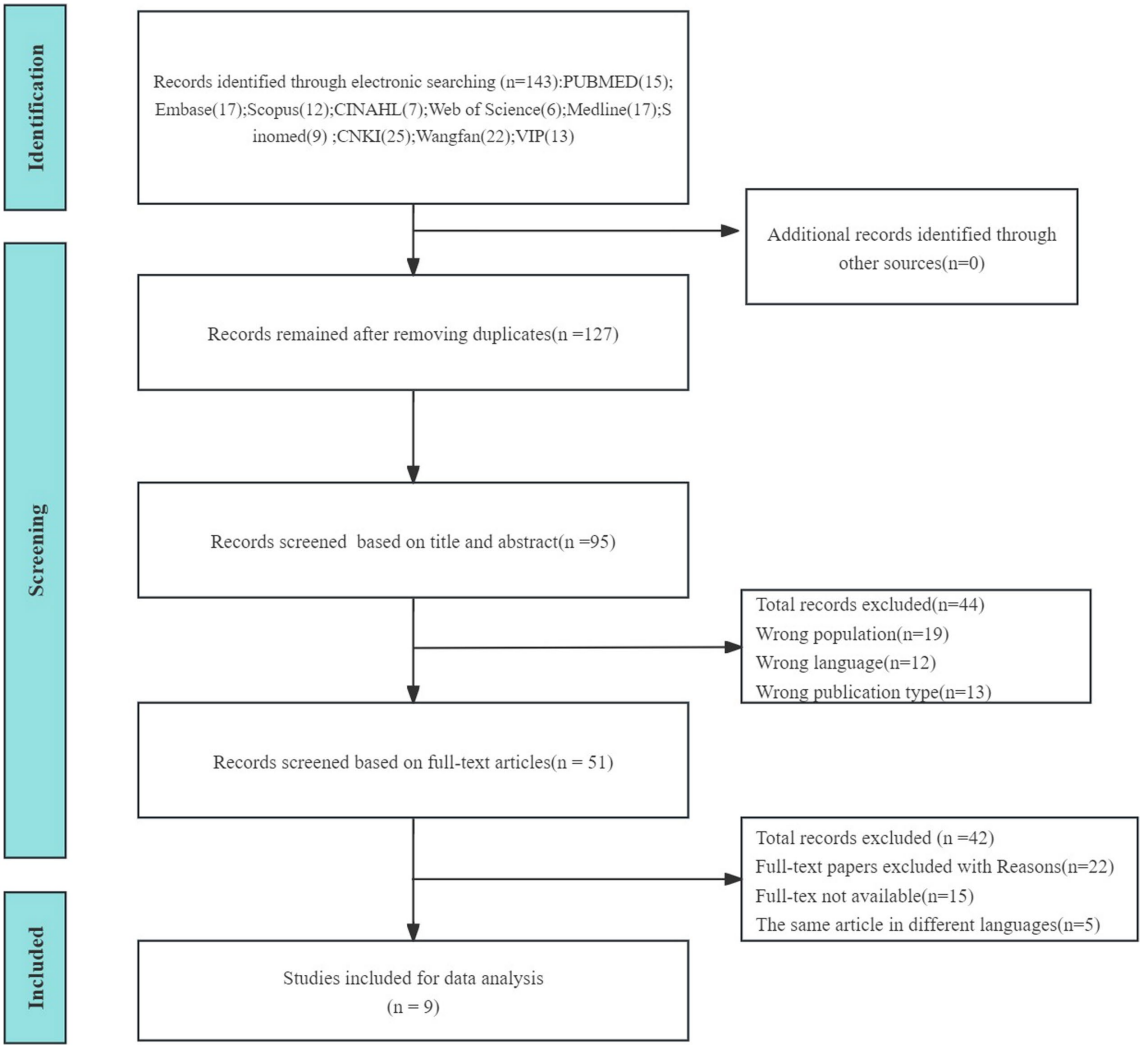
Flowchart of study retrieval process.

The titles of the study retrieved in the database were imported into EndNote 20 software, and after automatic and manual de-duplication, two trained researchers independently read the titles and abstracts of the study according to the inclusion and exclusion criteria of the study for the first screening. After the initial screening, the researchers further obtained the full text for the second screening. In the screening process, if the researchers had differences of opinion and could not reach a consensus, the third researcher was invited to participate in the discussion and resolution. In addition, the data extraction of the included study was also conducted by two researchers independently, and any discrepancies in the extraction process were resolved by discussion between the two researchers or adjudicated by a third researcher. Subsequently, the lead author conducted the study summary analysis, extracting key information such as author details, publication year, country, research methodology, sample size, incidence of kinesiophobia, influencing factors, and assessment tools. Following information extraction, researchers collaboratively developed data charts using Excel software tailored to the research inquiries. Throughout the study, descriptive statistics and narrative reviews were employed to organize and synthesize research findings (Stang, 2010; Zeng et al., 2012), with thematic analysis (Braun and Clarke, 2006) utilized to categorize influencing factors. See Figure 1 for more details.

## 3 Results

### 3.1 Descriptive statistics summary

A total of 143 relevant studies were preliminarily retrieved, and 9 studies were finally included (Hoffmann et al., 2018; Dai et al., 2022; Qin J. et al., 2022; Qin J-. W. et al., 2022; Chen and Li, 2023; Qin et al., 2023; Sentandreu-Mano et al., 2023; Tang et al., 2023; Zhang et al., 2023), with a total sample size of 1923 cases.

### 3.2 Current status of kinesiophobia in patients with chronic heart failure

Five studies specifically described the incidence of kinesiophobia in patients with chronic heart failure, ranging from 54.4 to 69.5%, among which Tang (Tang et al., 2023) had the highest incidence, indicating that patients with chronic heart failure had a high incidence of kinesiophobia. In terms of regions, 7 studies (Dai et al., 2022; Qin J. et al., 2022; Qin J-. W. et al., 2022; Chen and Li, 2023; Qin et al., 2023; Tang et al., 2023; Zhang et al., 2023) were from China, to a certain extent, the research on kinesiophobia in patients with chronic heart failure is not enough. The studies are summarized in Table 1.

**Table 1:** Basic characteristics of the included studies.

Author/Year	Country	Study type	Sample size	Incidence rate (%)	Age	Evaluation tools	Kinesiophobia score	Influencing factors
Chen and Li (2023)	China	Cohort studies	181	-	61.36 ± 12.57	TSK-Heart-C	On admission: 46.69 ± 7.83; 1 month after discharge: 43.15 ± 9.20; 3 months after discharge: 39.78 ± 7.53; 6 months after discharge: 34.21 ± 5.95	(1) (2) (3) (11) (18)
Dai et al. (2022)	China	Cross-sectional study	188	-	54.66 ± 16.49	TSK-SV Heart	34.88 ± 13.25	(2) (4) (5) (12) (13)
Qin J-. W. et al. (2022)	China	Cross-sectional study	260	65.00	76.80 ± 7.40	TSK-Heart-C	-	(4) (11) (14) (16) (18)
Sentandreu-Mano et al. (2023)	Spain	Cross-sectional study	107	54.40	73.18 ± 12.68	TSK-11	-	(1) (3) (16) (17)
Hoffmann et al. (2018)	Germany	Cross-sectional study	132	-	67.10 ± 12.10	TSK-SV Heart	-	(8) (9) (15) (19)
Qin et al. (2022)	China	Cross-sectional study	236	63.14	>60	TSK-Heart-C	59.31	(4) (5) (11) (17)
Tang et al. (2023)	China	Cross-sectional study	244	69.50	64.45 ± 11.076	TSK-SV Heart	-	(4) (5) (6) (12) (19)
Qin et al. (2023)	China	Cross-sectional study	270	-	76.17 ± 7.96	TSK-SV Heart-C	-	(10) (14) (18)
Zhang et al. (2023)	China	Cross-sectional study	305	58.40	64.41 ± 8.38	TSK-SV Heart	39.45 ± 76.91	(6) (7) (8)

Influencing factors: (1)Gender; (2) Age; (3) Social Support; (4) Educational Attainment; (5) Economic Status; (6) Occupation; (7) Social Status; (8) Anxiety and Depression; (9) Disease Awareness; (10) Coping Mechanisms; (11) Disease Progression; (12) Cardiac Function; (13) Surgical History; (14) Heart Failure Symptoms; (15) Comorbidities; (16) Fatigue; (17) Pain Perception; (18) Self-Efficacy; (19) Physical Exercise. Evaluation tools: (1) TSK-Heart-C: Chinese version of Kinesiophobia of Heart Disease Scale; (2) TSK-SV Heart: Kinesiophobia Scale for patients with heart disease; (3) TSK-11: Kinesiophobia of Heart Disease-11.

### 3.3 The influencing factors of kinesiophobia in people with chronic heart failure

After a systematic retrieval process, we meticulously sorted and synthesized the pertinent influencing factors gleaned from the included studies. These factors predominantly fall within four overarching categories, amounting to a total of 19 distinct influencers. See Table 2 for details.

**Table 2:** Influencing factors of kinesiophobia in CHF.

Theme	Specific factors
Socio-demographic factors	Gender, Age, Social Support, Educational Attainment, Economic Status, Occupation, Subject Social Status
Psychological and cognitive factors	Anxiety and Depression, Disease Awareness, Coping Mechanisms, Self-Efficacy
Disease and treatment factors	Disease Progression, Cardiac Function, Surgical History, Heart Failure Symptoms, Comorbidities, Fatigue, Pain Perception
Lifestyle and behavior	Physical Exercise

#### 3.3.1 Socio-demographic factors

Existing studies have shown that gender, age and other related socio-demographic factors of patients with chronic heart failure can affect the occurrence of kinesiophobia. In the retrieved studies, Chen and Li (2023) underscored the female gender as a noteworthy risk factor for fostering apprehension towards exercise among CHF patients (*p* < 0.05). Conversely, Acar et al. (2016) observed a higher incidence of exercise-related fear in men compared to women. Despite the contrasting findings, it is evident that gender plays a role in influencing fear of exercise. Age was also a risk factor for kinesiophobia (*p* < 0.05), and the older the age (Dai et al., 2022; Chen and Li, 2023), the higher the level of kinesiophobia. Social support was also an influencing factor of kinesiophobia. Studies (Chen and Li, 2023; Sentandreu-Mano et al. (2023) pointed out that the level of social support was negatively correlated with kinesiophobia, that is, the higher the level of social support, the lower the level of kinesiophobia (r = −0.48, *p* < 0.01). Education attainment was closely related to the level of kinesiophobia. Studies (Dai et al., 2022; Qin J. et al., 2022; Qin J-. W. et al., 2022; Tang et al., 2023) pointed out that the lower the education attainment, the higher the level of kinesiophobia, and the patients with junior high school education or below had a higher level of kinesiophobia (*p* < 0.05). Qin et al. (2022) found that educational background had the greatest impact on kinesiophobia [OR = 3.369, 95%CI (1.249–9.091)]. Patients with low economic income had a higher level of kinesiophobia (Dai et al., 2022; Qin J-. W. et al., 2022; Tang et al., 2023) than those with higher income (*p* < 0.05). Occupational status and type of occupation were the influencing factors of the occurrence of kinesiophobia, and two studies (Tang et al., 2023; Zhang et al., 2023) showed that non-working status (retired, unemployed or unemployed), workers, and farmers had higher levels of kinesiophobia (*p* = 0.002).

#### 3.3.2 Psychological and cognitive factors

Two studies (Hoffmann et al., 2018; Zhang et al., 2023) pointed out that kinesiophobia was related to anxiety (r = 0.418, *p* < 0.05) and depression (r = 0.498, *p* < 0.05). On the one hand, a high level of kinesiophobia was positively correlated with disability rate and depression in patients with heart disease; on the other hand, the occurrence of kinesiophobia could also lead to anxiety in patients. Patients with CHF frequently experience psychological challenges stemming from the prolonged disease trajectory and escalating physical symptoms (Zhang et al., 2019). In such instances, cardiophobia often surfaces, representing an anxiety disorder characterized by recurrent physical discomfort like chest pain and palpitations, coupled with an intense fear of heart attack and mortality, despite the absence of underlying physical pathology (Eifert, 1992). The level of disease awareness (Hoffmann et al., 2018) and coping mechanisms (Qin et al., 2023) were also risk factors for kinesiophobia (*p* < 0.01). Three studies (Qin J-. W. et al., 2022; Chen and Li, 2023; Qin et al., 2023) pointed out that self-efficacy was related to kinesiophobia (*p* < 0.01). The lower the level of self-efficacy, the higher the level of kinesiophobia.

#### 3.3.3 Disease and treatment factors

Disease progression was positively correlated with the level of kinesiophobia (Qin J. et al., 2022; Qin J-. W. et al., 2022; Chen and Li, 2023), and the longer the disease progression, the greater the likelihood of heightened kinesiophobia levels (*p* < 0.05). Two studies (Dai et al., 2022; Tang et al., 2023) showed that the level of cardiac function classification was also positively correlated with the level of kinesiophobia (*p* < 0.01). Another study pointed out that the history of surgery (*p* < 0.05) (Dai et al., 2022); Heart failure symptoms (Qin J-. W. et al., 2022; Qin et al., 2023) such as dyspnea and activity intolerance (*p* < 0.01). Heart failure symptoms and comorbidities are closely related to kinesiophobia (Hoffmann et al., 2018; Qin J-. W. et al., 2022; Qin et al., 2023; r=0.44, *p* <0.01). The more serious the symptoms of heart failure and the more the number of comorbidities, the higher the level of kinesiophobia in CHF patients. Studies (Qin J. et al., 2022; Qin J-. W. et al., 2022) showed that fatigue was negatively correlated with kinesiophobia (r = 0.49, *p* < 0.01). Fatigue manifests as a persistent subjective sensation affecting the body, emotions, or cognition. Patients typically experience negative moods, diminished energy, and disrupted rest (Bower, 2014). Moreover, heightened levels of fatigue correlate with increased kinesiophobia among patients. One study (Sentandreu-Mano et al., 2023) showed that the location, intensity, and frequency of pain perception were related to kinesiophobia, especially patients with knee and waist pain had higher levels of kinesiophobia (*p* < 0.01). Exercise avoidance due to pain is the primary cause of kinesiophobia (Lethem et al., 1983). Patients who excessively focus on pain and threatening stimuli will develop fear and resistance towards physical activities, leading to exercise avoidance and impeding the rehabilitation process.

#### 3.3.4 Lifestyle and behavior

Studies (Hoffmann et al., 2018; Tang et al., 2023) pointed out that kinesiophobia was related to the physical exercise of patients with heart failure (*p* < 0.05).

### 3.4 Kinesiophobia assessment tools

In the 9 included studies, a total of 3 kinesiophobia assessment tools were utilized: TSK-Heart-C, TSK-SV Heart, and TSK-11.

#### 3.4.1 The Tampa Scale for Kinesiophobia heart

The scale, adapted and developed by the Bäck (Bäck et al., 2012) from the Tampa Scale for Kinesiophobia (TSK). Is first used to measure the level of kinesiophobia heart patients. The scholars of different countries, respectively, to cross-cultural adaptation and the scale reliability and validity of test. The TSK-SV Heart scale has 17 items and is divided into 4 dimensions: risk perception, kinesiophobia, exercise avoidance and dysfunction. The total score ranged from 17 to 68 points, and >37 points were considered to have kinesiophobia, and the higher the score, the more serious the degree of kinesiophobia.

#### 3.4.2 Tampa Scale for Kinesiophobia

It was originally used to assess fear of exercise-related pain in patients with musculoskeletal pain (Miller et al., 1991). The Cronbach’s α coefficient of each version of TSK was between 0.7 and 0.92, and the test–retest reliability was above 0.8, which had good reliability and validity. Kamonseki (Zale and Ditre, 2015) translated the Short Form for Kinesiophobia Assessment (TSK-11) into a Brazilian version and applied it to patients with shoulder pain.

## 4 Discussion

The current situation of kinesiophobia in patients with chronic heart failure is not optimistic. According to existing studies, the level of kinesiophobia in patients with chronic heart failure reaches 54.40–69.50% (Qin J. et al., 2022; Qin J-. W. et al., 2022; Sentandreu-Mano et al., 2023; Tang et al., 2023; Zhang et al., 2023). The current research in this field is mainly concentrated in China, with less research in other regions, which to some extent reflects the insufficient attention paid to CHF-related anxiety in other regions. Our research shows that the current studies are mostly cross-sectional surveys, lacking comprehensive policy guidance or targeted interventional studies. Our study indicates that the assessment tools for CHF-related anxiety are mostly subjective questionnaires, lacking objective assessment indicators or tools, which highlights the importance of medical and nursing staff assessing the level of CHF-related anxiety in a timely manner and developing suitable objective assessment tools in the future, which will improve the accuracy of assessment and promote the achievement of cardiac rehabilitation for CHF patients.

The study review conducted in this study revealed 19 factors that affect kinesiophobia in patients with CHF, which can be categorized into four thematic categories. Among them, the socio-demographic factors and disease and treatment factors themes contain the most factors, with 7 factors each. It is worth noting that although our study categorized the factors affecting kinesiophobia in patients with CHF into four themes, each theme is not independent and can interact with each other. The factors of social support in the socio-demographic factors domain and comorbidity factors in the disease and treatment domain are closely associated with mental health. Social support encompasses emotional, informational, and tangible aid from various sources such as family, friends, and the community (Langford et al., 1997). Effective social support can significantly alleviate patients’ psychological distress, including depression (Patra et al., 2017). Additionally, an increase in the number of complications can elevate the risk of depression (Wiltink et al., 2011). Social support can also enhance patients’ access to medical information. Patients who heavily rely on social support tend to leverage the social resources available to them to acquire disease-related information through various channels (Qiao et al., 2021). Furthermore, social support directly influences self-efficacy (Liu et al., 2023). Patients with high self-efficacy are confident enough to actively engage in postoperative rehabilitation training, thereby establishing and adopting healthy behaviors, enhancing compliance with treatment, and developing skills for health self-management to promote cardiac rehabilitation.

Among all these factors, educational attainment has proven to be the most prevalent influencing factor, indicating a significant effect on kinesiophobia. Patients with higher levels of education demonstrate greater abilities in acquiring disease-related information and self-management skills (Qiao et al., 2021). This, in turn, empowers them to make informed decisions. Conversely, CHF patients with lower educational backgrounds may lack a comprehensive understanding of their condition, potentially leading to fear of early functional exercise and resistance to early rehabilitation training. Consequently, the optimal rehabilitation window may be missed, thereby impacting patient rehabilitation. This underscores the importance of giving increased attention to CHF patients with lower educational backgrounds in clinical settings. Implementing targeted interventions or health education based on the educational level of CHF patients holds significant value.

Low-income CHF patients often face significant financial burdens, worrying that engaging in physical activity may exacerbate their condition and escalate medical costs. Thus, they often resort to avoidance and passive coping strategies in managing daily activities and functional exercises. However, employment not only offers financial stability but also fosters social interaction, structure, and a sense of accomplishment, all of which can alleviate symptoms of kinesiophobia.

Social status is a position within society determined by an individual or group’s access to resources, including income, education, occupation, and other factors (Bradley and Corwyn, 2002). It encompasses both objective measures and subjective perceptions, with subjective social status more accurately influencing life attitudes, behaviors, and future expectations (Goodman et al., 2003). Subject social status (Zhang et al., 2023) exhibited a negative correlation with kinesiophobia (*p* < 0.05). Higher social status affords greater resources, social recognition, and influence, thereby enhancing individual well-being and mental health (Adler et al., 2000).

Coping style serves as a cognitive behavioral strategy individuals employ to effectively manage internal and external stressors. Patients adopting a positive coping style are more likely to exhibit psychological resilience to adverse reactions, thereby optimizing health outcomes and improving quality of life. Conversely, individuals inclined towards negative responses may lack confidence and succumb to ineffective outcomes, exacerbating health consequences. Patients with multiple comorbidities often exhibit reduced tolerance for self-exercise and are prone to avoidant coping, leading to diminished motivation for physical activity. Consequently, patients with lower activity levels are more susceptible to developing kinesiophobia. Despite the myriad benefits regular activity and exercise offer, such as enhanced physical functioning, improved sleep quality, and better management of negative emotions (Chakravarty et al., 2008), individuals grappling with kinesiophobia often fail to fully leverage these advantages. This limitation, in part, presents a challenge to cardiac rehabilitation in CHF patients.

While existing studies have highlighted associations between social status, disease awareness, coping mechanisms, surgical history, comorbidities, and kinesiophobia in CHF patients, further research is needed to deepen our understanding of these factors. Future studies should comprehensively explore these aspects to inform effective intervention measures. Accurate assessment of exercise fear in heart failure patients is pivotal for promoting exercise rehabilitation. Although various versions of exercise fear assessment scales exist and have been applied in diverse populations, researchers must carefully select the most suitable research tool considering regional and population characteristics to accurately gauge the level of exercise fear in future studies.

In general, the 9 studies (Hoffmann et al., 2018; Dai et al., 2022; Qin J. et al., 2022; Qin J-. W. et al., 2022; Chen and Li, 2023; Qin et al., 2023; Sentandreu-Mano et al., 2023; Tang et al., 2023; Zhang et al., 2023) included in this study can reflect the influencing factors of the occurrence of kinesiophobia in patients with chronic heart failure, and provide a basis for the development of clinical targeted nursing measures. However, only part of the studies have conducted follow-up, which may cause bias in the study results. Secondly, on the issue of kinesiophobia, most of the studies included in this study were analyzed from the patients themselves, and there were certain limitations in the perspective of discussion. In addition, kinesiophobia is a dynamic process, and long-term follow-up of patients is needed to evaluate the impact of kinesiophobia on cardiac rehabilitation. To effectively reduce or mitigate the fear of movement in patients with CHF, a comprehensive approach is necessary, encompassing multidisciplinary professional interventions and governmental policy support, particularly for economically disadvantaged patients. Given the chronic nature of CHF and its prolonged treatment process, patients often bear a substantial disease burden, making appropriate medical subsidies or relief crucial. Hence, future research should adopt a multifaceted approach, examining various perspectives, including those factors or populations currently underrepresented. A thorough exploration of targeted interventions for kinesiophobia is paramount. Additionally, further research is warranted to investigate the long-term effects of kinesiophobia on the cardiac rehabilitation outcomes of CHF patients.

## 5 Limitations

The reviewers encountered challenges related to language barriers, resulting in the consideration of only published Chinese or English. There may also be a certain degree of selection bias present. The reviewers did not reach out to the researchers involved in this study for additional relevant information, potentially leading to some missing data. Furthermore, the methodological quality of the studies included was not systematically evaluated. Lastly, the assessment instrument utilized in these studies consisted of a self-report questionnaire and lacked objective measures of kinesiophobia, suggesting potential bias in the reported levels.

## 6 Conclusion

Patients with chronic heart failure have a high incidence of kinesiophobia and many influencing factors, but the current research is not comprehensive. In the future, more clinical interventions can be further carried out based on the results of existing research to reduce the incidence of kinesiophobia in patients with chronic heart failure and promote the realization of cardiac rehabilitation based on exercise rehabilitation.

## Data availability statement

The original contributions presented in the study are included in the article/Supplementary material, further inquiries can be directed to the corresponding author.

## Author contributions

QX: Writing – original draft, Writing – review & editing. X-YX: Conceptualization, Funding acquisition, Supervision, Writing – review & editing. M-JZ: Data curation, Formal analysis, Writing – review & editing. SL: Data curation, Formal analysis, Resources, Writing – original draft. HC: Methodology, Supervision, Writing – review & editing. M-DL: Data curation, Formal analysis, Writing – review & editing. YW: Methodology, Resources, Software, Writing – review & editing. YY: Resources, Supervision, Writing – review & editing.
