# Supplementary material for: Incidence and influencing factors of kinesiophobia in patients with chronic heart failure: a scoping review
Source: Front Psychol. 2024 Jul 31;15:1395199. doi: 10.3389/fpsyg.2024.1395199 (PMC11322567; doi:10.3389/fpsyg.2024.1395199)
Supplement: Supplementary file 1 [file Table_1.DOCX]

**Appendix 1: Search strategy**

**Search conducted on December 20, 2023**

| **Search** | **Query** |
| --- | --- |
| #1 | ("heart failure"[MeSH Terms]) |
| #2 | ((((((("chronic heart failure"[Title/Abstract]) OR ("CHF"[Title/Abstract])) OR ("cardiac failure*"[Title/Abstract])) OR ("heart decompensation"[Title/Abstract])) OR ("right-side heart failure"[Title/Abstract])) OR ("myocardial failure"[Title/Abstract])) OR ("congestive heart failure"[Title/Abstract])) OR ("left-side heart failure"[Title/Abstract]) |
| #3 | #1 OR #2 |
| #4 | ("kinesiophobia"[Mesh]) |
| #5 | ("fear of movement"[Title/Abstract]) OR ("fear of physical activity"[Title/Abstract]) |
| #6 | #4 OR #5 |
| #7 | ("risk factors"[MeSH Terms]) |
| #8 | ((((((("relative risk"[Title/Abstract]) OR ("social risk factor*"[Title/Abstract])) OR ("health correlate*"[Title/Abstract])) OR ("population at risk"[Title/Abstract])) OR ("risk score"[Title/Abstract])) OR ("risk factor score*"[Title/Abstract])) OR ("cause"[Title/Abstract])) OR ("protective factor"[Title/Abstract]) |
| #9 | #7 OR #8 |
| #10 | #3 AND #6 AND #9 Filters: CHINESE or ENGLISH language |
